# Insulin alleviates the inflammatory response and oxidative stress injury in cerebral tissues in septic rats

**DOI:** 10.1186/1476-9255-11-18

**Published:** 2014-06-20

**Authors:** Qiyi Chen, Wenkui Yu, Jiangliang Shi, Juanhong Shen, Tao Gao, Juanjuan Zhang, Fengchan Xi, Jieshou Li, Ning Li

**Affiliations:** 1 Department of General Surgery, Jinling Hospital, Medical School of Nanjing University, 305 East Zhongshan Road, Nanjing 210002, Jiangsu Province, China

**Keywords:** Sepsis, Sepsis-associated encephalopathy, Inflammation, Oxidative stress, Neurologic injury

## Abstract

Sepsis-associated encephalopathy (SAE) is a diffuse brain dysfunction that occurs secondary to infection in the body without overt central nervous system (CNS) infection. SAE is frequently encountered in critically ill patients in intensive care units and can be detected in up to 50–70% of septic patients. Previous studies have demonstrated that inflammatory cytokine release and oxidative stress injury are major pathophysiological mechanisms of SAE in critically ill patients. However, there are no effective strategies for the treatment of SAE. Insulin has important immunomodulatory effects and protective effects against oxidative stress injury in the peripheral organs of septic patients. However, very few studies of the possible effects of insulin in cerebral tissues of septic patients have been reported. Therefore, in this study, we aimed to explore whether insulin therapy can inhibit cytokine production (IL-1, IL-6, and TNF-a) and oxidative stress injury of the brain tissue in septic rats. We observed that the protein concentrations of IL-1, IL-6, and TNF-а, in addition to MDA and H_2_O_2_ were notably increased, inversely SOD, and GSH were sigificantly decreased in cortex, hippocampus, and hypothalamus of septic rats. Furthermore, the levels of S100 and NSE significantly increased. After 6 hours of insulin therapy, we found that the cytokine concentrations notably decreased and oxidative stress injuries in the cortex, hypothalamus, and hippocampus were alleviated in septic rats. In addition, the S100 and NSE levels significantly decreased. We concluded that insulin can inhibit the production of inflammatory cytokines and the oxidative stress response, thereby improving brain tissue damage.

## Introduction

**Figure 1:**
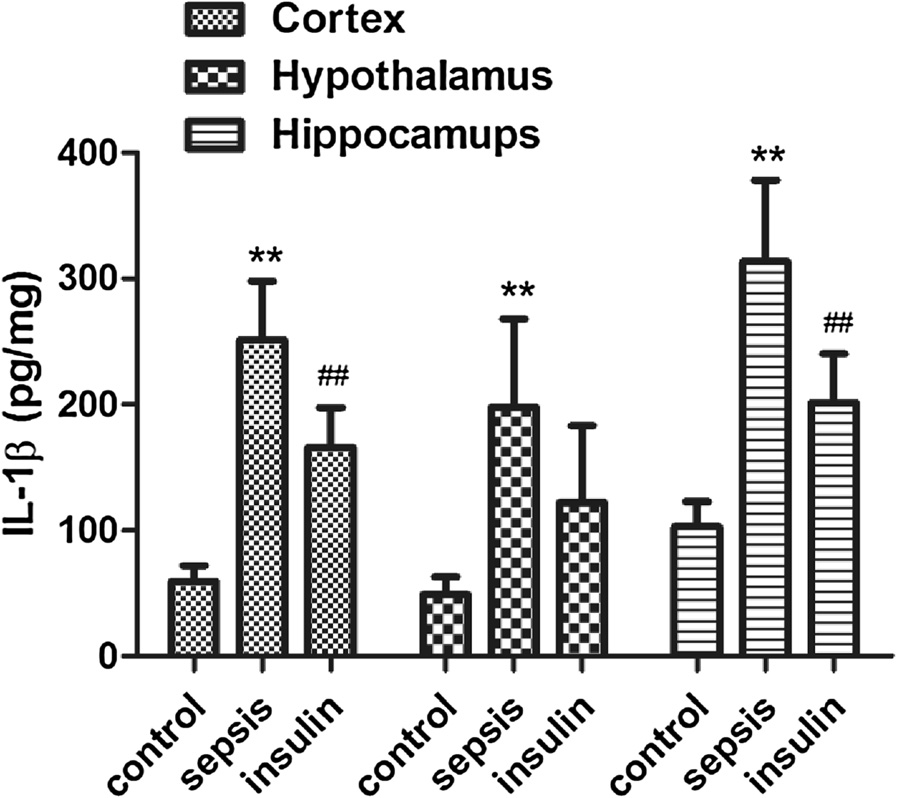
**Effect of insulin (4.8 mu/kg/min) on LPS (10 mg/kg) induced changes in IL-1β in different brain regions (cortex, hypothalamus and hippocampus).** Value are expressed as mean +/- SD of each group (n=6). Compared with control group, **p<0.01, compared with sepsis group, ##p<0.01.

**Figure 2:**
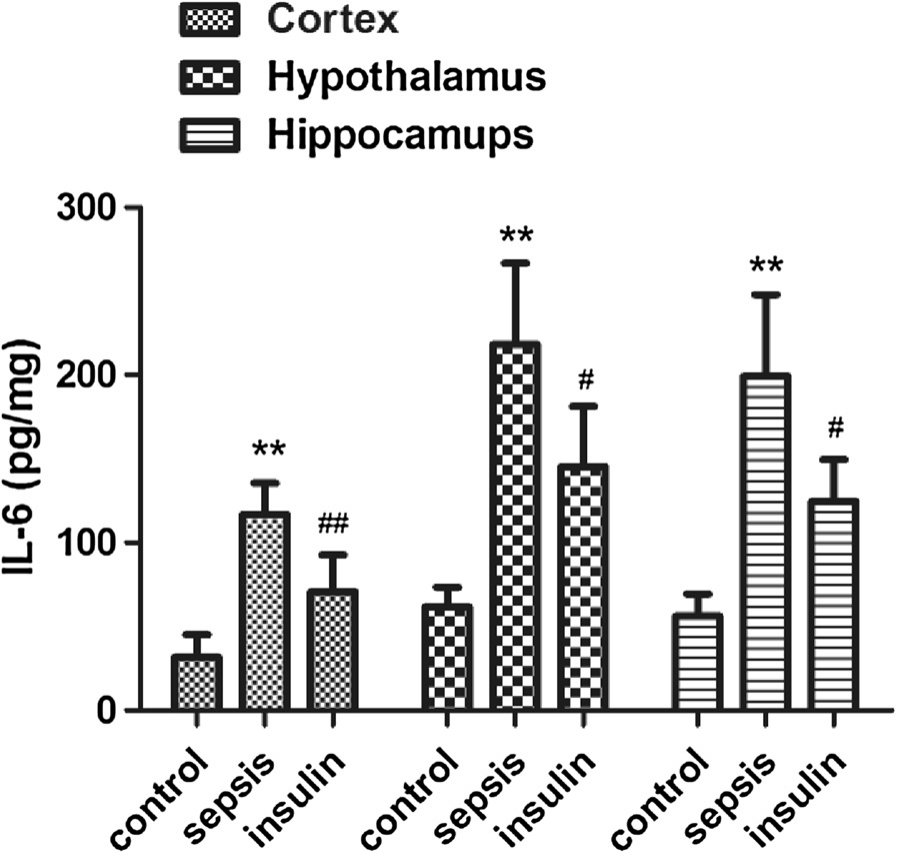
**Effect of insulin (4.8 mu/kg/min) on LPS (10 mg/kg) induced changes in IL-1β in different brain regions (cortex, hypothalamus and hippocampus).** Value are expressed as mean +/- SD of each group (n=6). Compared with control group, **p<0.01, compared with sepsis group, #p<0.05, ##p<0.01.

**Figure 3:**
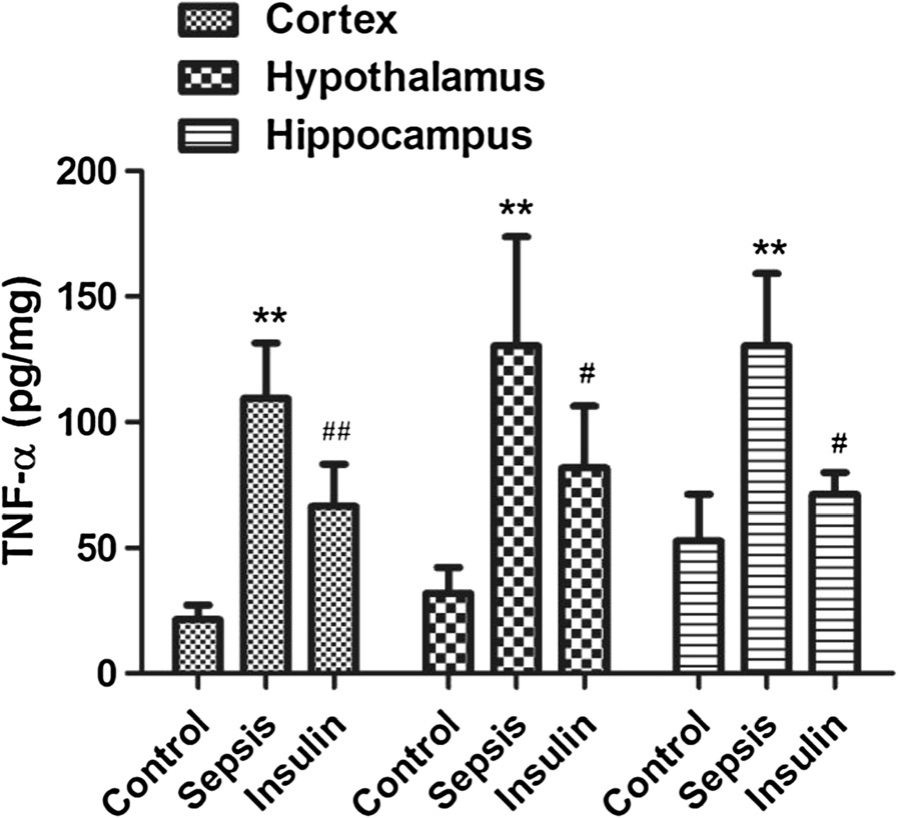
**Effect of insulin (4.8 mu/kg/min) on LPS (10 mg/kg) induced changes in TNF-α in different brain regions (cortex, hypothalamus and hippocampus).** Value are expressed as mean +/- SD of each group (n=6). Compared with control group, **p<0.01, compared with sepsis group, #P<0.05, ##p<0.01.

**Figure 4:**
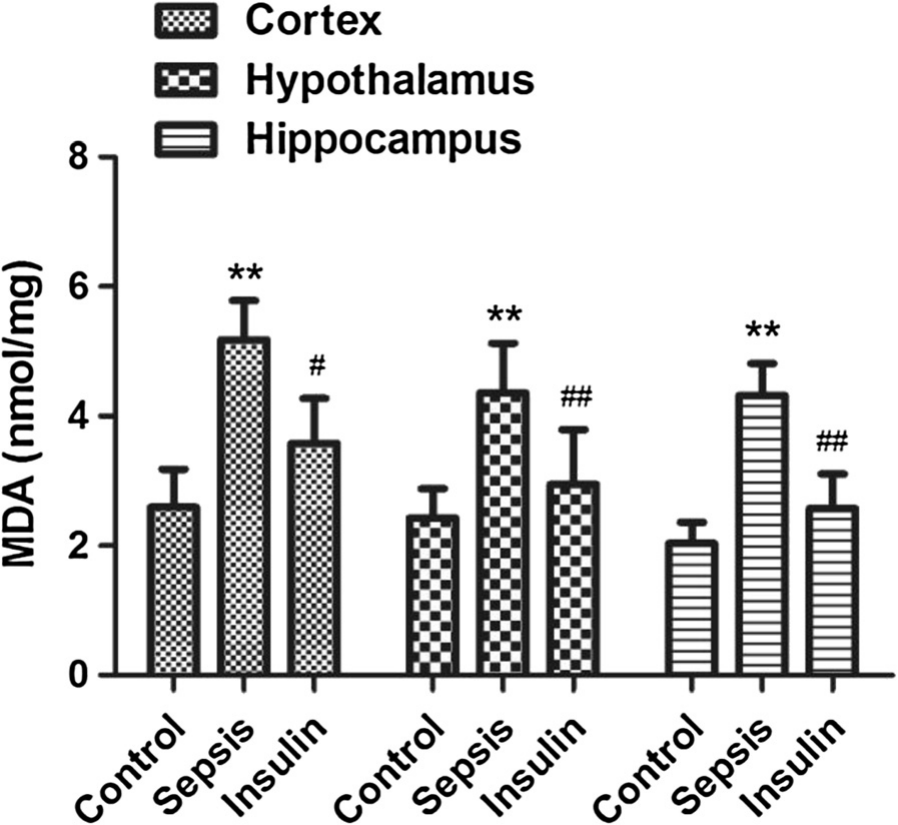
**Effect of insulin (4.8 mu/kg/min) on LPS (10 mg/kg) induced changes in MDA in different brain regions (cortex, hypothalamus and hippocampus).** Value are expressed as mean +/- SD of each group (n=6). Compared with control group, **p<0.01, compared with sepsis group, #P<0.05, ##p<0.01.

**Figure 5:**
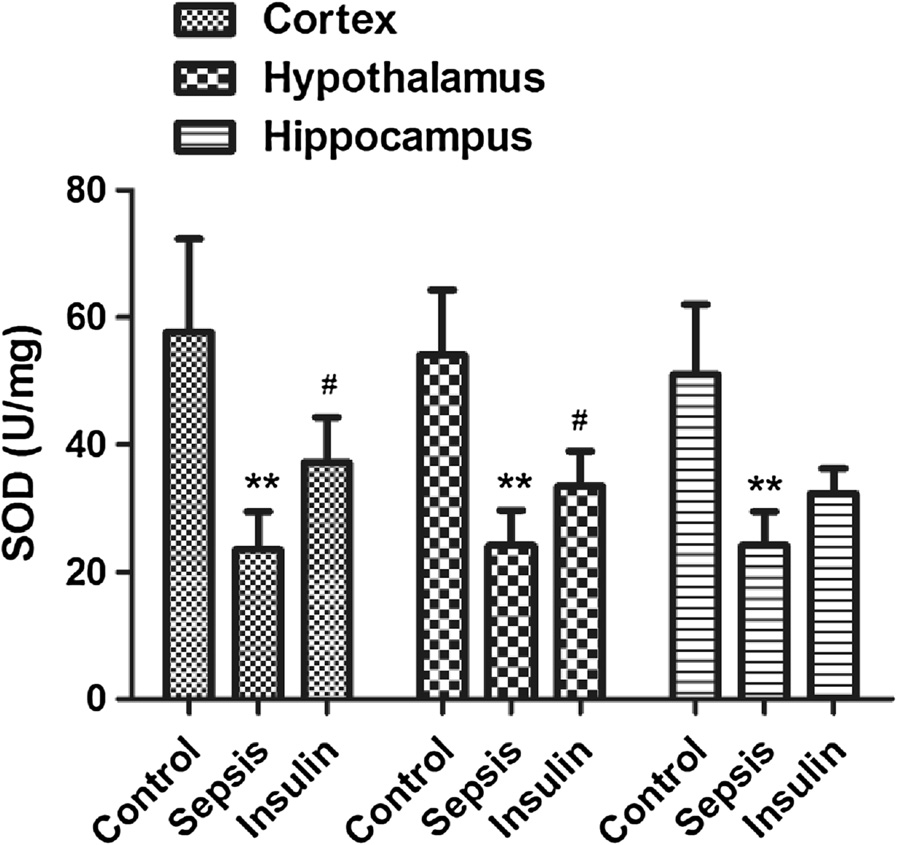
**Effect of insulin (4.8 mu/kg/min) on LPS (10 mg/kg) induced changes in SOD in different brain regions (cortex, hypothalamus and hippocampus).** Value are expressed as mean +/- SD of each group (n=6). Compared with control group, **p<0.01, compared with sepsis group, #p<0.05.

**Figure 6:**
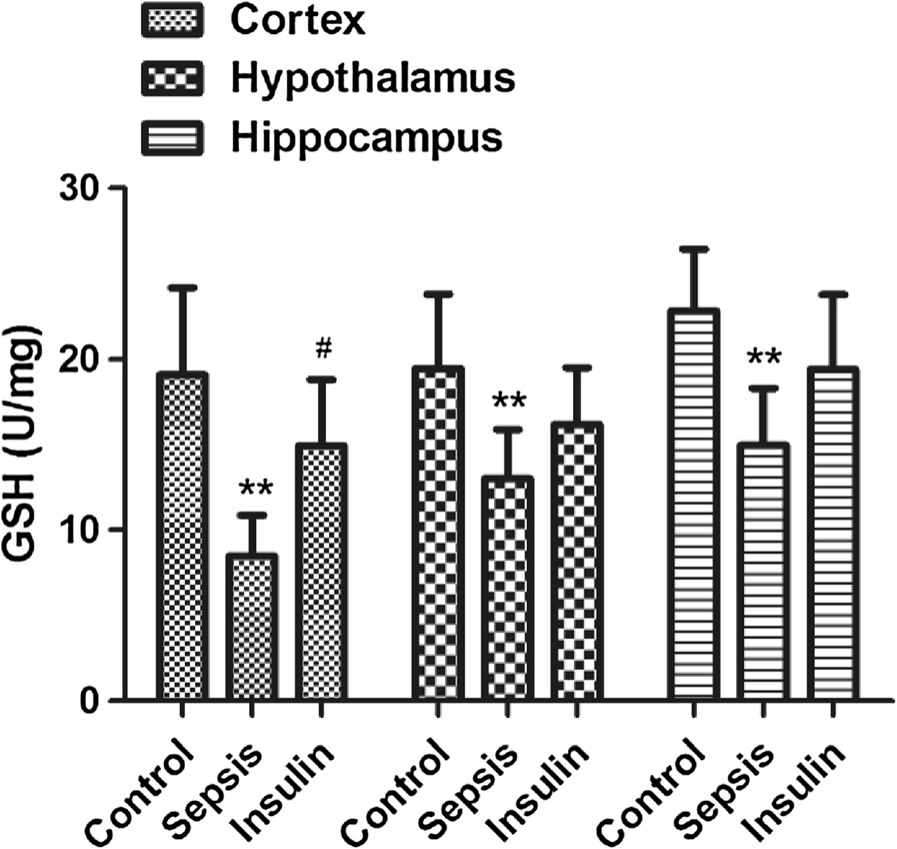
**Effect of insulin (4.8 mu/kg/min) on LPS (10 mg/kg) induced changes in GSH in different brain regions (cortex, hypothalamus and hippocampus).** Value are expressed as mean +/- SD of each group (n=6). Compared with control group, **p<0.01, compared with sepsis group, #p<0.05.

**Figure 7:**
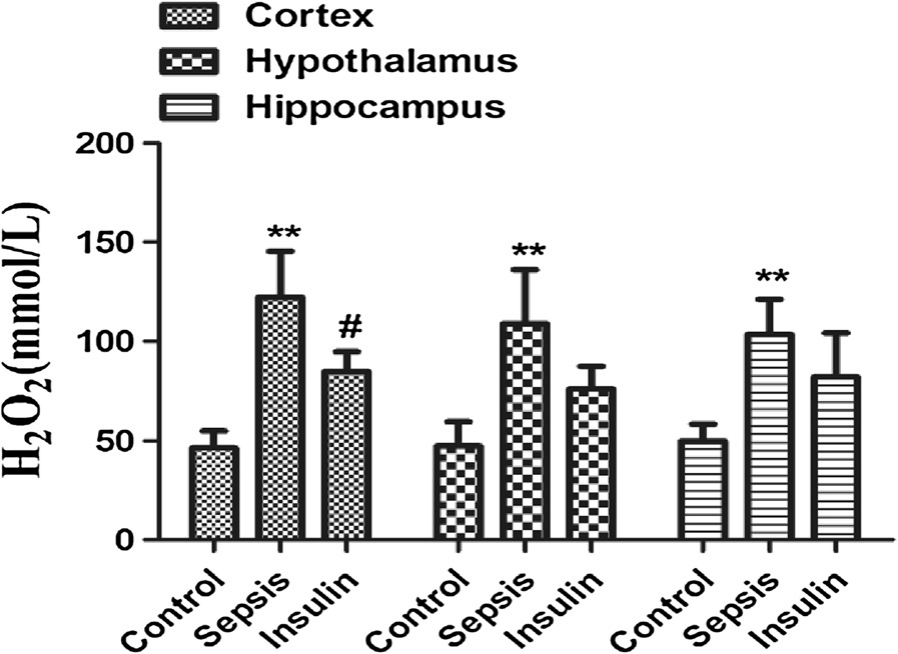
**Effect of insulin (4.8 mu/kg/min) on LPS (10 mg/kg) induced changes in H**_**2**_**O**_**2**_**in different brain regions (cortex, hypothalamus and hippocampus).** Value are expressed as mean +/- SD of each group (n=6). Compared with control group, **p<0.01, compared with sepsis group, #p<0.05.

**Figure 8:**
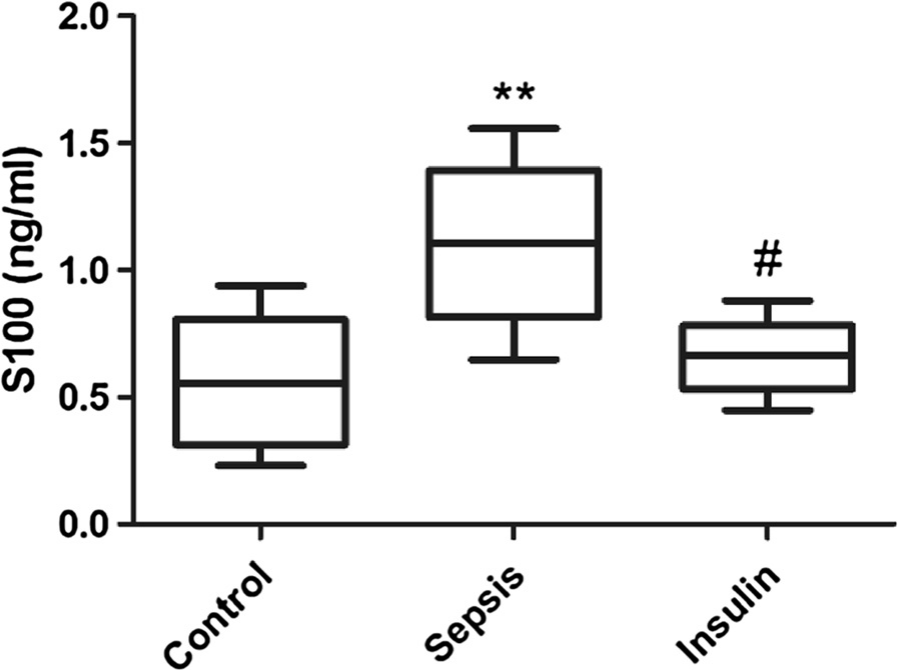
**Effect of insulin (4.8 mu/kg/min) on LPS (10 mg/kg) induced changes in S100 in serum.** Value are expressed as mean +/- SD of each group (n=6). Compared with control group, **p<0.01, compared with sepsis group, #p<0.05.

**Figure 9:**
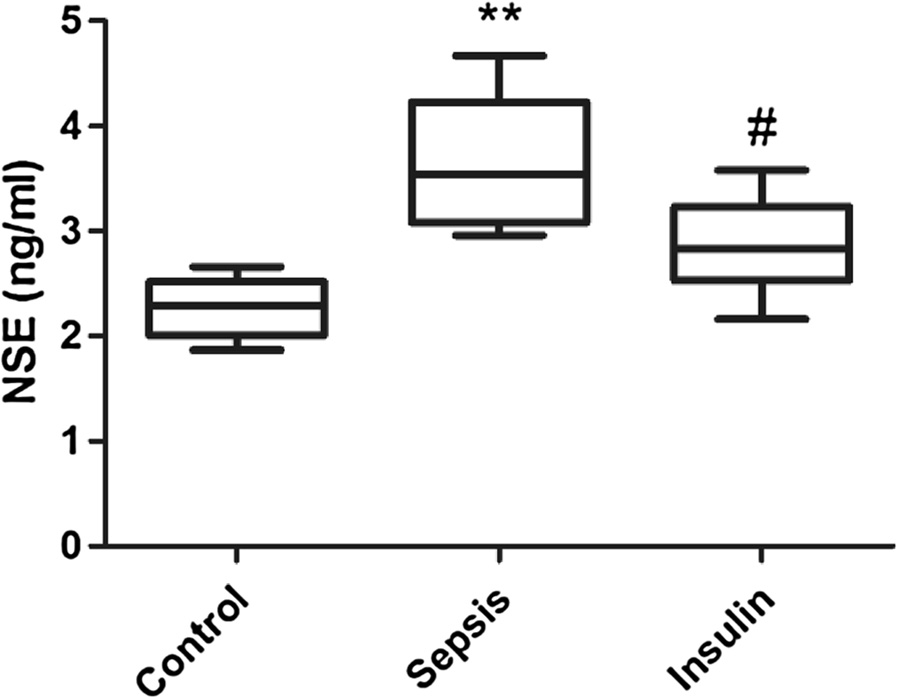
**Effect of insulin (4.8 mu/kg/min) on LPS (10 mg/kg) induced changes in NSE in serum.** Value are expressed as mean +/- SD of each group (n=6). Compared with control group, **p<0.01, compared with sepsis group, #p<0.05.

Sepsis-associated encephalopathy (SAE) is a diffuse brain dysfunction that occurs secondary to infection in the body in the absence of central nervous system (CNS) infection. SAE is often observed in critically ill patients and can be detected in up to 50–70% of septic patients [1,2]. However, there are no effective treatment strategies for SAE. The severity of SAE can range from mild delirium to deep coma. Patients with acutely altered mental status associated with encephalopathy have higher mortality rates (49%) than patients with pre-existing mental status changes (41%) or a normal mental status (26%) [1] (Figures 1, 2, 3, 4, 5, 6, 7, 8 and 9).

Previous studies have established that inflammatory cytokine release and oxidative stress injury are major pathophysiological mechanisms of sepsis, resulting in injury to system tissues and organs [3,4]. Further studies have shown that the mechanisms underlying sepsis are related to cytokine generation and oxidative stress injury [3,5]. These effects can have several deleterious consequences, including nerve cell apoptosis and necrosis [6-8], neuronal bioenergetic failure and cerebral oxidative metabolism injury [9], axonal injury and brain tissue edema [10,11], neurotransmitter transporter inhibition [12], and destruction of the blood–brain barrier [7]. A number of studies have confirmed that anti-inflammatory cytokines and reduction in oxidative stress have significant protective effects against brain injury [8,13-17]. Therefore, the inhibition of inflammation and oxidative stress can prevent brain tissue injury and ameliorate SAE.

In recent years, controversies have arisen regarding insulin therapy in critically ill patients. In particular, the results of 2 milestone investigations conducted between 2001 and 2009 showed contradictory mortality benefits [18,19]. There are conflicting opinions on whether insulin therapy itself, in addition to the modification of glucose levels (regulated by insulin), is suitable for critical care patients. Insulin therapy provides better controllability and maneuverability in animal experiments and therefore has obvious benefits in animal models. In addition to the regulation of glucose levels, insulin therapy has important immunomodulatory effects in septic patients. Previous studies conducted by our group and others have demonstrated that insulin therapy can directly inhibit the release of cytokines such as IL-1, IL-6, and TNF-a under septic conditions [4,20]. Furthermore, it can also inhibit the transcription factor NF-κB with subsequent downregulation of proinflammatory cytokines [21]. In addition, insulin can suppress oxidative stress by ameliorating mitochondrial function or inhibiting the release of cytokines in septic patients and animals [21,22].

At present, studies of insulin therapy for critical care subjects focus on peripheral tissues, and very few studies assessing whether insulin can influence cerebral tissues of septic patients have been reported. Therefore, in this study, we aimed to explore whether insulin therapy can inhibit the production of cytokines (IL-1, IL-6, and TNF-a) and oxidative stress injury of the brain tissue in septic rats. Our findings may provide a theoretical basis for the clinical treatment of SAE.

## Materials and methods

### Animals

In this study, we used 54 adult male Sprague–Dawley rats, weighing 280 ± 20 g, from the animal center of Jinling Hospital. The Institutional Animal Care Committee approved the study protocol. The Association accredits the animal care facility for Assessment and Accreditation of Laboratory Animal Care. Rats were housed in mesh cages in a 25°C room, illuminated in 12-h light: 12-h dark cycles. The animals were allowed to acclimatize to their environment for 7 d before the start of the study. They were provided with standard rodent chow and water *ad libitum*. The rats were fasted for 12 h immediately before the experiment.

### Animal preparation

Rats were anesthetized intraperitoneally with phenobarbital sodium (60 mg/kg). Catheters (PE-50, PE-10; Becton-Dickinson, Sparks, MD) were implanted into the right femoral vein for the infusion of insulin and dextrose solutions via a micropump (provided by the Research Center for Analytical Instrument, Zhejiang University). The catheters were filled with saline containing heparin sodium. About 1 mm of the tail tip was cut for monitoring blood glucose levels. The scab of the incision was abscised with 75% alcohol every time. The area around the incision was gently squeezed and the first drop of blood was discarded; the next drop was used for monitoring using an Elite glucometer (Bayer, Elkhart, IN).

### Group distribution and insulin infusion strategy

After the animals were prepared for the experiments, they were divided randomly into 3 groups as follows: control group (n = 12), LPS group (n = 12), and insulin treatment group (n = 12). The sepsis model was mimicked by intraperitoneal injection of 10 mg/kg LPS (*Escherichia coli* serotype 055:B5; Sigma, St. Louis, MO). The rats in the insulin treatment group received a continuous infusion of insulin (Humulin R, Eli Lilly & Co., Indianapolis, IN) at a constant rate of 4.8 mU · min^-1^ · kg^-1^ for 6 h after LPS stimulation. Blood glucose was maintained at 140–180 mg/dL by varying the infusion rate of a 50% dextrose solution. The LPS group was injected intraperitoneally with 10 mg/kg LPS only. The control group received an intraperitoneal injection of an equal volume of sterile saline only. The rats remained anesthetized for the duration of the experiment by the continuous infusion of phenobarbital sodium.

At the end of the infusion, the rats were killed with an overdose of phenobarbital sodium. The frontal cortex, hippocampus, and hypothalamus were dissected and stored at -80°C until analysis.

### Determination of protein concentrations of inflammatory cytokines in cerebral tissues

To determine the protein concentrations of TNF-a, IL-1β, and IL-6 in cerebral tissues (cortex, hypothalamus, and hippocampus), the tissues were diluted (40% w/v) with 0.01 mol/L phosphate-buffered saline, pH 7.4, containing a protease inhibitor cocktail (Roche, Indianapolis, IN), and homogenized. The homogenates were then centrifuged at 7,500 × *g* for 20 min at 4°C. The levels of TNF-a, IL-1β, and IL-6 in the supernatant were determined by enzyme-linked immunosorbent assay performed using commercial kits (R&D Systems, Minneapolis, MN) in accordance with the manufacturer’s instructions. The results are expressed as pg/mg of brain tissue.

### Measurements of antioxidants and oxidant indices in brain tissues

The tissue homogenates were utilized for the determination of antioxidants (SOD and GSH) and oxidant indices (MDA) using commercial analysis kits (Nanjing Jiancheng Bioengineering Institute, China). SOD and GSH activities of the brain tissues (cortex, hypothalamus, and hippocampus; n = 6/region/group) are expressed in terms of U/mg protein, and MDA concentrations are expressed in nmol/mg (n = 6/group).

### Measurements of H_2_O_2_ in brain tissues

The tissue homogenates were utilized for the determination of H_2_O_2_ using commercial analysis kits (Nanjing Jiancheng Bioengineering Institute, China). The concertrations of H_2_O_2_ in brain tissue (n = 6/regions/group) were expressed in terms of mmol/L.

### Detection of serum S100 and neuron-specific enolase

Blood samples were collected from the femoral vein at 6 h after insulin infusion for the measurement of S100 and neuron-specific enolase (NSE) levels. The blood was allowed to clot for 20–30 min at room temperature and then centrifuged and frozen at temperatures lower than -70°C. Serum S100 and NSE levels were quantified with an enzyme-linked immunosorbent assay (R&D, USA) according to the manufacturer’s instructions.

### Statistical analysis

Data are expressed as mean ± standard deviation (SD). Statistical analysis was performed using ANOVA. All data were analyzed using the SPSS software (Statistical Package for the Social Sciences, version 20.0, for Windows, SPSS, Chicago, IL). A p value < 0.05 was considered significant.

## Results

### Variation in the protein concentrations of the cytokines IL-1β, IL-6, and TNF-a in cerebral tissues

The protein concentrations of the cytokines IL-1β, IL-6, and TNF-a in the cortex, hypothalamus, and hippocampus significantly increased after intraperitoneal injection of 10 mg/kg LPS (vs. control group, p < 0.01). When insulin was injected at 4.8 mU · kg^-1^ · min^-1^ for 6 h, the IL-6 and TNF-a levels decreased significantly in the cortex, hypothalamus, and hippocampus (vs. sepsis group, p < 0.05). Although the IL-1 level decreased only mildly in the hypothalamus (122.11 ± 61.03 vs. 198 ± 70.49 in the sepsis group, p = 0.21), it significantly decreased in the cortex and hippocampus (vs. sepsis group, p < 0.05).

### Variation in antioxidant and oxidant indices in cerebral tissues

After injection of 10 mg/kg LPS, the MDA concentrations in all brain regions (cortex, hypothalamus, and hippocampus) notably increased (vs. control group, p < 0.01), but the GSH and SOD activities in all brain regions significantly decreased (vs. control group, p < 0.01). When insulin was injected at 4.8 mU · kg^-1^ · min^-1^ for 6 h, IL-6 and MDA levels decreased significantly in all brain regions (vs. sepsis group, p < 0.05). SOD and GSH activities were upregulated to different degrees in all brain regions; SOD upregulation was most obvious in the cortex and hypothalamus (vs. sepsis group, p < 0.05), whereas GSH upregulation was most significant in the cerebral cortex (vs. sepsis group, p < 0.05).

### Variation in ROS system indices H_2_O_2_ in cerebral tissues

The H_2_O_2_ concentrations in all brain regions were notably increased (vs. control group, p < 0.01), after injection of 10 mg/kg LPS. When insulin was injected by 4.8 mU/(kg∙min) for 6 hours, we found that H_2_O_2_ were decreased sigificantly in cortex (vs. sepsis group, p < 0.05). and the H_2_O_2_ were also decreased moderatly in hypothalamus and hippocampu, although both no significant difference (vs. sepsis group, p > 0.05).

### Variation in the serum protein levels of S100B and NSE

The concentrations of S100 and NSE in the serum notably increased (vs. control group, p < 0.01) after injection of 10 mg/kg LPS. However, when insulin was injected at 4.8 mU · kg^-1^ · min^-1^ for 6 h, the serum S100 and NSE levels significantly decreased (vs. sepsis group, p < 0.05).

## Discussion

During sepsis, the brain may be one of the first organs affected [23,24]. SAE, a diffuse brain dysfunction, can be detected in up to 50–70% of septic patients, and patients with acute SAE have a high mortality rate (49%) [1,2]. Several lines of evidence indicate that an inflammatory cascade and oxidative stress injury are the main mechanisms of SAE. However, at present, the establishment of therapeutic strategies for the treatment of SAE in clinical practice is problematic.

Under septic conditions, the expression of cytokines (e.g., IL-1, IL-6, TNF-а, and IL-10) is significantly upregulated in cerebral tissues. In agreement with this, our results show that the protein concentrations of the cytokines IL-1, IL-6, and TNF-а notably increased in the cortex, hippocampus, and hypothalamus of septic rats. Numerous studies suggest that cytokines cause brain toxicity. The reported injurious effects of cytokines include nerve cell apoptosis and necrosis [6-8], neuronal bioenergetic failure and cerebral oxidative metabolism injury [9], axonal injury and brain tissue edema [10,11], neurotransmitter transporter inhibition [12], and destruction of the blood–brain barrier [7].

An imbalance between oxidants and antioxidants in favor of the oxidants, potentially leading to damage, is termed “oxidative stress”. Oxidants are formed as a normal product of aerobic metabolism, but they can also be produced at elevated rates under pathophysiological conditions [25]. The antioxidant systems in the body include SOD, glutathione peroxidase (GSH-Px), catalase, and GSH [26,27]. MDA is the main oxidation product of peroxidized polyunsaturated fatty acids, and an increased MDA level is an important indication of lipid peroxidation [26,27]. The brain is particularly susceptible to oxidative stress because of its high metabolic rate, relatively low capacity for cellular regeneration [28], and low antioxidant capacity due to a lack of reduced GSH [29-31]. During sepsis, oxidative stress injury is the main pathophysiological mechanism of SAE. Our experimental results showed that the levels of the antioxidants SOD and GHS notably decreased in septic rats in every region of the brain (hippocampus, hypothalamus, and cortex), whereas the MDA levels notably increased. These results are similar to previous observations that an imbalance exists between oxidants and antioxidants during sepsis.

Reactive oxygen species (ROS) including superoxide $O_{2}^{-}$, hydrogen peroxide (H_2_O_2_), and hydroxyl radicals are generated in both normal and pathological, biological processes. ROS system can significantly were activated and participated in the pathophysiology process of sepsis [32]. The study found similar, in sepsis, H_2_O_2_ was increased significantly. The present study found that H_2_O_2_ significantly reduced the GPx, SOD, and CAT activities, while MDA level exposed to H_2_O_2_ was elevated,indicating disruptions of the endogenous antioxidant enzymes [33]. Agents that inhibit the production of reactive oxygen species or increase the antioxidant defense may prevent apoptosis and protect cells from oxygen radicals damage [33]. Insulin can prevent mitochondrial generation of H_2_O_2_ in normal rat neuronal cultures [34]. In our results showed H_2_O_2_ were decreased in cortex, hypothalamus and hippocampus in different degree when insulin was injected by 4.8 mU · kg^-1^ · min^-1^ for 6 hours.

Above-mentioned results show that insulin can inhibit inflammatory cytokines and the oxidative stress response. The interaction between cytokines and oxidative stress has also been recently investigated. Cytokines have been reported to increase the neutrophil oxidative respiratory burst [35]; however, oxidative stress can be an initiator of cytokine release and cell damage [36]. Therefore, with respect to SAE, it might be useful to inhibit the inflammatory response and correct the imbalance between oxidants and antioxidants in brain tissues. In addition to the regulation of blood glucose levels, insulin plays important roles in immune regulation and the inhibition of oxidative stress injury. Our previous studies and a number of other reports have demonstrated that insulin can significantly reduce the release of inflammatory cytokines and improve the prognosis of critically ill patients [20]. To date, studies of insulin therapy for critical care subjects have focused on peripheral tissues, and the effects of insulin in the cerebral tissues of septic patients have not been thoroughly investigated. Therefore, in our experiment, we infused insulin intravenously at 4.8 mU · kg^-1^ · min^-1^ and maintained the blood glucose level at 140–180 mmol/dL by intravenous infusion of a 50% dextrose solution. After 6 h of insulin therapy, we found that cytokine concentrations notably decreased and oxidative stress injury in the cortex, hypothalamus, and hippocampus was alleviated in septic rats.

Serum S100B and NSE are specific biomarkers of cerebral injury [37,38]. S100B is most abundant in glial cells of the CNS, mainly in astrocytes, while NSE is present almost exclusively in the cytoplasm of neurons (γ–γ isoenzyme) and neuroendocrine cells (α–γ isoenzyme). Therefore, in our next study, we will further examine the serum content of S100 and NSE. In the present study, we found that the serum levels of S100 and NSE were notably increased after injection of 10 mg/kg LPS. However, when insulin was injected at 4.8 mU · kg^-1^ · min^-1^ for 6 h, we found that the serum levels of S100 and NSE significantly decreased. Therefore, the results of this study indicate that insulin can inhibit cerebral injury.

In conclusion, our results show that insulin can inhibit inflammatory cytokines and the oxidative stress response and consequently improve brain tissue damage. The findings of this study may provide a basis for the development of treatment strategies for SAE in clinical practice.

## Abbreviations

SAE: Sepsis-associated encephalopathy; CNS: Central nervous system; NSE: Neuron-specific enolase.

## Competing interests

The authors declare that they have no competing interests.

## Authors’ contributions

QC, JShi and JShen participated in the collection of data. NL and WY conceived and designed this study. TG, JZ and FX performed the statistical analysis. QC, JShi and JShen wrote the first draft of the paper and JL commented on the draft. All the other authors provided comments and approved the final manuscript. All authors read and approved the final manuscript.
